# Effects of *Periploca chevalieri* Browicz on Postprandial Glycemia and Carbohydrate-Hydrolyzing Enzymes

**DOI:** 10.3390/ph18060913

**Published:** 2025-06-18

**Authors:** Katelene Lima, Maryam Malmir, Shabnam Sabiha, Rui Pinto, Isabel Moreira da Silva, Maria Eduardo Figueira, João Rocha, Maria Paula Duarte, Olga Silva

**Affiliations:** 1 Research Institute for Medicines (iMed.ULisboa), Faculty of Pharmacy, Universidade de Lisboa, 1649-003 Lisbon, Portugal; k.lima@edu.ulisboa.pt (K.L.); m.malmir@edu.ulisboa.pt (M.M.); s.sabiha@edu.ulisboa.pt (S.S.); rapinto@ff.ulisboa.pt (R.P.); icsilva@ff.ulisboa.pt (I.M.d.S.); efigueira@ff.ulisboa.pt (M.E.F.); jrocha@ff.ulisboa.pt (J.R.); 2 Dr Joaquim Chaves Laboratório de Análises Clínicas, 2790-224 Carnaxide, Portugal; 3 The Mechanical Engineering and Resource Sustainability Center (MEtRICs), Chemistry Department, Nova School of Science and Technology, Universidade Nova de Lisboa, 2829-516 Caparica, Portugal; mpcd@fct.unl.pt

**Keywords:** *α*-amylase, *α*-glucosidase, antiglycation, diabetes, DPP4, herbal medicine, *Periploca chevalieri*

## Abstract

**Background/Objectives:** *Periploca chevalieri* Browicz (*Apocynaceae*), an endemic species of the Cabo Verde archipelago, is commonly used in traditional medicine for the treatment of diabetes. The aim of this study was to characterize the chemical profiles of the aqueous and hydroethanolic (70%) extracts of the *P. chevalieri* dried aerial parts (PcAE and PcEE) and evaluate their potential to modulate postprandial glycemia and inhibit key carbohydrate-hydrolyzing enzymes. **Methods:** The chemical characterization was performed by LC/UV-DAD-ESI/MS/MS. An in vivo evaluation of postprandial glycemia modulation was conducted on healthy CD1 mice submitted to an oral sucrose tolerance test. In vitro enzymatic inhibition was performed for the α-amylase, α-glucosidase, and DPP4 enzymes. Additionally, antioxidant and antiglycation activities were also assessed. **Results:** Phenolic acid derivatives, flavanols, proanthocyanidins, and flavonols were the major classes of secondary metabolites identified. PcEE at 170 mg/kg of body weight significantly (*p* < 0.05) reduced the postprandial glycemia peak in CD1 mice submitted to sucrose overload. Regarding the enzymatic inhibition, both extracts showed concentration-dependent inhibitory potential against the α-amylase, α-glucosidase, and DPP4 enzymes. Both extracts inhibited α-glucosidase more effectively than acarbose. **Conclusions:** The obtained results supports the traditional use of *P. chevalieri* and suggest the potential for further pharmacological investigation.

## 1. Introduction

Diabetes mellitus is a serious health problem, ranking as the eighth main global cause of death in 2021 [1]. According to the International Diabetes Federation, 11.1% (589 million) of the world’s adult population (20–79 years) was living with diabetes in 2024 and this number is expected to increase to 13% (853 million) by 2050 [2,3]. In Africa, 25 million people (1 in 20 adults) have diabetes, and the prediction is that by 2050, the number will increase 142%, reaching 60 million people [2].

Diabetes mellitus is an epidemic chronic metabolic disorder characterized by persistent hyperglycemia resulting from inadequate insulin secretion and/or the development of insulin resistance [4,5,6]. It is classified into two main types: type 1, which is caused by impaired insulin secretion, and type 2, which is mainly associated with the inability of cells to respond to insulin (i.e., insulin resistance) [5,7]. Type 2 diabetes mellitus (T2DM) is the most common type of diabetes mellitus, accounting for more than 90% of all cases worldwide [2].

Nowadays, several modern medicines and therapies options are available for the management of T2DM and diabetes complication. These treatments are able to improve insulin sensitivity (e.g., metformin and thiazolidinediones), increase insulin secretion (sulfonylureas and insulin secretagogues (e.g., meglitinides)), decrease the plasma glucose concentration (inhibitors of sodium-glucose co-transporter-2 (SGLT2)), decrease glucose absorption (*α*-glucosidase inhibitors), and increase insulin secretion by targeting the incretin system (incretin agonists and dipeptidyl peptidase 4 (DPP4) inhibitors) [8,9]. Furthermore, when these therapies do not provide adequate glycemic control, subcutaneous insulin is indicated as a therapeutic option for T2DM [10].

Nevertheless, the incidence of T2DM continues to increase worldwide along with a substantial economic burden, complaints of side effects, and a lack of compliance with the available therapeutics [2,5,7,11,12]. Additionally, T2DM is a considerable cause of polypharmacy, explained by the necessity to treat the microvascular and macrovascular complications, but also due to the presence of several comorbidities [13]. In this sense, the treatment of T2DM has seen a shift from monotherapy to combination therapy in the mode “multi-drugs to multi-targets.”

Medicinal plants with antidiabetic potential are being evaluated as alternative therapies in the management of the multifactorial etiology of diabetes and its associated complications. As complex chemical mixtures containing secondary metabolites with multiple potential targets and mechanisms of action [14], medicinal plants and herbal medicinal products may be promising agents for the management of T2DM. In fact, several plants and herbal medicinal products and their bioactive secondary metabolites have demonstrated substantial antidiabetic potential while causing minimal or no serious adverse reactions [15] through in vitro, in vivo, and clinical studies [16,17,18].

The antihyperglycemic actions of medicinal plants are associated with their ability to modulate glucose metabolism through different pathways, such as restoring β-cell integrity, enhancing insulin-releasing activity, and increasing cellular glucose uptake, which can improve insulin resistance [19,20,21]. Additionally, they can contribute to the control of the complications associated with T2DM at different levels, providing antioxidant compounds that act against oxidative stress and protein glycation [22,23].

*Periploca chevalieri* Browicz, an endemic species of the Cabo Verde archipelago [24,25,26], belongs to the family *Apocyanaceae* Juss. [27] and to subfamily *Periplocoideae* [28]. *Periploca* is one of the largest genera of *Periplocoideae* subfamily [29], with 17 accepted species according to Plants of the World [27]. The genus is distributed throughout semiarid habitats of Northeastern Africa and Western Asia, including the Arabian Peninsula [29,30]. The therapeutic properties of several *Periploca* species (*Periploca sepium* Bunge, *Periploca forresti* Schltr., *Periploca graeca* L., *Periploca angustifolia* Labill., and *Periploca laevigata* Aiton) have been reported in the traditional medicine of several countries. They have been used to treat several health problems, including cardiovascular diseases such as hypertension [31], inflammation (swellings and rheumatoid arthritis) [32], as well as diabetes [33,34].

In Cabo Verde, *P. chevalieri*, commonly known as “lantisco” (Santo Antão, Santiago and Fogo Islands) and “curcabra” (São Nicolau Island), is used to treat cough and diabetes and as a depurative and cardiotonic agent [35,36,37].

Over the years, several authors have studied the genus *Periploca* and reported an array of pharmacological activities and chemical compounds. Cardenolides, C_21_ pregnane-type steroids, phytosterols, triterpenoids, phenylpropanoids, flavonoids, quinones, aromatic acids, and carbohydrates were the most predominant chemical compounds identified in the studied species [32]. The pharmacological potential of some of these compounds has also been studied and has been associated with specific activities, such as cardiotonic effects due to the presence of periplocin; anti-inflammatory activity due to the presence of periplosides, periplocin, together with several terpenoids; and antioxidant activity related to 4-methoxysalicylaldehyde, lignins, flavans, and polysaccharides [32].

To date, no scientific studies have reported the chemical composition or biological activity of *Periploca chevalieri*. This study addressed that gap by characterizing the profile of secondary metabolites present in *Periploca chevalieri* aerial parts and evaluating their potential to modulate postprandial glycemia, with the aim of scientifically validating their ethnopharmacological use.

## 2. Results

### 2.1. Chemical Studies

#### 2.1.1. Drug–Extract Ratio (DER)

The drug–PcAE ratio was 15:1 and the drug–PcEE ratio was 2.9:1. The yield for PcAE was found to be 6.6% and 40.1% for PcEE.

#### 2.1.2. HPLC/UV-DAD-ESI/MS/MS Chemical Profile

**Figure 1:**
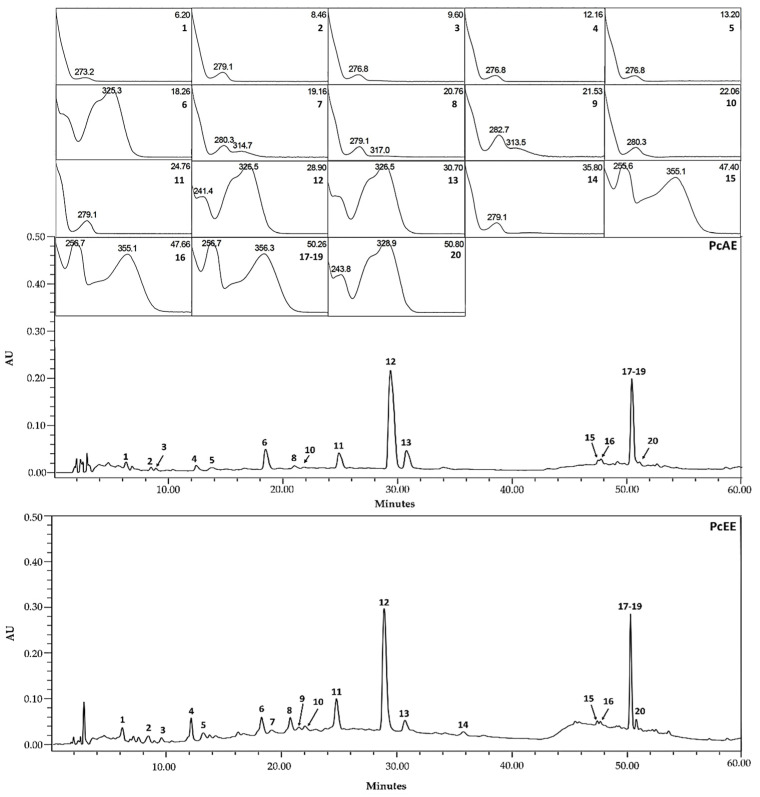
LC/UV-DAD *maxplot* chromatogram profile and UV spectra with retention times of the *Periploca chevalieri* aqueous extract (PcAE) and *Periploca chevalieri* hydroethanolic extract (PcEE).

**Figure 2:**
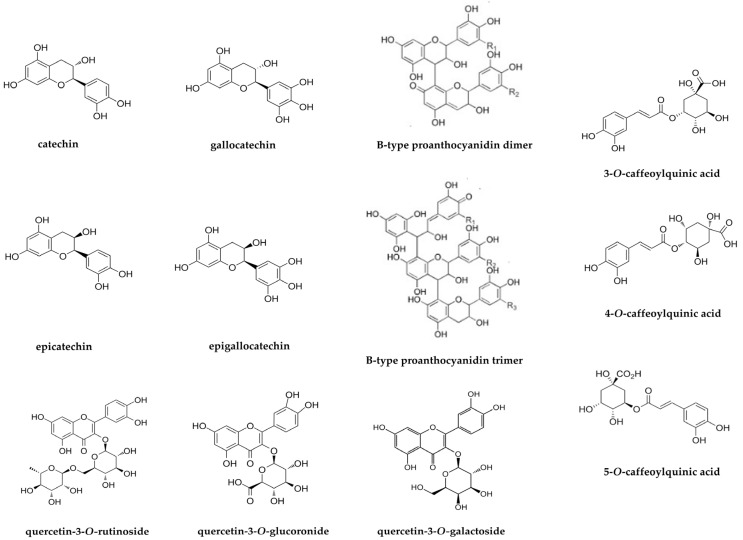
Main marker secondary metabolite structures from *P. chevalieri aerial* part extracts.

The main marker compounds detected in *P. chevalieri* extracts were tentatively identified by a comparison of the retention time (*t*_R_), wavelength of maximum absorbance (*λ*max), molecular ion ([M−H]^−^ or [M+H]^+^), and corresponding fragment ions (MS^n^) with those of reference standards and/or published data in the literature (Figure 1, Table 1). Twenty marker compounds were detected and categorized as phenolic acids, flavan-3-ols, proanthocyanidins, and flavanols (Figure 2).

Peaks 1, 6, 12, 13, and 20 were identified as phenolic acids; peaks 2, 10, 11, and 14 were identified as flavan-3-ols; peaks 3, 4, 5, 7, 8, and 9 were identified as proanthocyanidins; and peaks 15, 16, 17, 18, and 19 were identified as flavonols (Table 1).

Peak 1 with a *λ*_max_ of 273.2 nm and a precursor ion [M−H]^−^ at *m*/*z* 191 was tentatively identified as quinic acid. Characteristic fragments at *m*/*z* 85 [M-H-2CO_2_-H_2_O]^−^, *m*/*z* 93 [M-H-CO_2_-3H_2_O]^−^, and *m*/*z* 127 [M-H-CO_2_-H_2_O-2H]^−^ were assigned to the losses of water (18 Da) and carbon dioxide (44 Da) moieties [38,39].

Peaks 6, 12, and 13, with *λ*_max_ of 325.3, 241, and 326.5, respectively, and the same precursor ion [M−H]^−^ at *m*/*z* 353 generated the same characteristic fragments at *m*/*z* 191 [quinic acid-H]^−^, 179 [caffeoyl-H]^−^, 173 [quinic acid-H-H_2_O]^−^, and 135 [caffeoyl-H-CO_2_]^−^, suggesting the presence of chlorogenic acid isomers [40]. The differences in the relative abundances of the three major product ions, namely, *m*/*z* 191, 179, and 173, allowed the differentiation between the three isomers according to the study by Willems et al. 2016 [41] regarding to the differentiation of monocaffeoylquinic acids by MS/MS. With a fragmentation pattern characterized by two major peaks at *m*/*z* 191 and *m*/*z* 179, peak 6 was identified as 3-*O*-caffeoylquinic acid (neochlorogenic acid), while peak 13 with a major base peak at *m*/*z* 191 and relatively minor product ions at *m*/*z* 179 and 173 was identified as 5-*O*-caffeoylquinic acid (chlorogenic acid). Peak 12 was differentiated from the other two isomers by presenting a base peak at *m*/*z* 173 showing a higher intensity than the other base peaks at *m*/*z* 191 and 179 and was identified as 4-*O*-caffeoylquinic (cryptochlorogenic acid). Neochlorogenic acid and chlorogenic acid identities were also confirmed by co-chromatography with reference standards.

Peak 20 with a *λ*_max_ of 325 nm, a precursor ion [M−H]^−^ at *m*/*z* 515, and fragment ions at 353, 191, 179, and 173 was tentatively identified as a dicaffeoylquinic acid derivative, namely, 1,4-dicaffeoylquinic acid, due to the identical fragmentation pathway with that of 4-*O*-caffeoylquinic acid [40].

Peaks 2–5, 7–11, and 14 were tentatively identified as flavan-3-ol monomers and polymers (proanthocyanidins).

The typical fragmentation patterns of proanthocyanidin molecules correspond to Heterocyclic Ring Fission (HRF, neutral loss of 126 Da), retro-Diels–Alder (RDA) reactions (neutral loss of 168 Da and 152 Da), rearrangements (neutral loss of 140 and 168 Da), cleavage of the interflavan linkage and loss of water (neutral loss of 18 Da), and quinone methide (QM) fragmentations, which define two monomeric units [42,43].

Peaks 2 and 10 with *λ*_max_ of 279.1 nm and 280.3 nm, respectively, and a precursor ion [M−H]^−^ at *m*/*z* 305 were tentatively identified as (+)-gallocatechin and (−)-epigallocatechin, and peaks 11 and 14 with the same *λ*_max_ of 279.1 nm and same precursor ion [M−H]^−^ at *m*/*z* 289 were identified as (+)-catechin and (−)-epicatechin, respectively, by co-chromatography with reference standards. Peaks 2 and 10 generated the same characteristic fragment ions at *m*/*z* 125 and at *m*/*z* 167 [M-140]^−^ and *m*/*z* 137 [M-168]^−^ by RDA fragmentation and rearrangements [43,44].

Peaks 3 and 4 with *λ*_max_ of 276.8 nm and a precursor ion [M−H]^−^ at *m*/*z* 593, and peaks 7 and 8, with *λ*_max_ of 280.3 and 314.7 nm and 279.1 and 317 nm, respectively, and a precursor ion [M−H]^−^ at *m*/*z* 577 were tentatively identified as B-type proanthocyanidin dimers. The fragmentation pattern of peaks 3 and 4, with characteristic a fragment at *m*/*z* 289 indicating QM fragmentation and fragments at *m*/*z* 425 [M-H-168]^−^ and *m*/*z* 407 [M-H-168-18]^−^ indicating RDA fragmentation and the successive loss a water molecule, allowed their tentative identification as dimeric B-type proanthocyanidin isomers with (epi)catechin and (epi)gallocatechin as monomeric units. Peaks 7 and 8, which produced similar characteristic fragment ions at *m*/*z* 289 (QM fragmentation), *m*/*z* 425 [M-H-152]^−^, and *m*/*z* 407 [M-H-152-18]^−^ (RDA fragmentation), were proposed as dimeric B-type proanthocyanidin isomers with two (epi)catechins as monomeric units [43].

Peaks 5 and 9 with *λ*_max_ of 278 nm and 282.7 and 313.5 nm, and precursor ions [M−H]^−^ at *m*/*z* 897 and *m*/*z* 881, respectively, were tentatively identified as trimeric B-type proanthocyanidin isomers. Similar characteristic fragment ions at *m*/*z* 305 and at *m*/*z* 289 were generated by QM fragmentations, indicating the combination of three monomeric units: one (epi)catechin and two (epi)gallocatechin units (peak 5) and two (epi)catechin and one (epi)gallocatechin units (peak 9) [43].

Peaks 15 to 19 were tentatively identified as quercetin glycosides. The possible glycoside moieties linked to the aglycone quercetin were tentatively assigned as glucose or galactose (162 Da), rhamnoside (146 Da), rutinoside or neohesperidoside (308 Da), and arabinoside, arabinofuranoside, or xyloside (132 Da) [45].

Peaks 15 and 16 with *λ*_max_ of 255.6 and 355.1 nm and 256.7 and 355.1 nm and the same precursor ion [M+H]^+^ at 757 were tentatively identified as quercetin 3-*O*-(2″,6″-di-*O*-rhamnosyl) galactoside and quercetin 3-*O*-(2″,6″-di-*O*-rhamnosyl) glucoside, respectively. The characteristic fragment at *m*/*z* 303 was assigned to the aglycone quercetin after the loss of galactoside or glucose [M+H-162]^+^ molecules. The successive neutral loss of 146 Da generated fragments at *m*/*z* 611 [M+H-146]^+^ and at *m*/*z* 465 [M+H-146-146] ^+^, indicating the presence of two rhamnosyl moieties in the structure [45,46].

Peaks 17 and 19 with *λ*_max_ at 256.7 and 356.3 nm and precursor ions [M−H]^−^ at *m*/*z* 609 and 463 were identified as quercetin-3-*O*-rutinoside and quercetin-3-*O*-galactoside by co-chromatography with reference standards. Peak 17 generated characteristic fragments at *m*/*z* 301 and at *m*/*z* 300 corresponding to quercetin aglycone and aglycone radical ion.Peak 19 with a precursor ion [M−H]^−^ at *m*/*z* 463 generated fragments at *m*/*z* 301 and at *m*/*z* 300, which correspond to the aglycone quercetin and quercetin radical ion, after the loss of galactoside and galactoside radical, respectively [M-H-162]^−^. Other product ions at *m*/*z* 271, *m*/*z* 243, and *m*/*z* 227 were generated after the consecutive loss of COH, CO, and O from the ion at *m*/*z* 300. RDA cleavage of the aglycone resulted in characteristic ions at *m*/*z* 151 and *m*/*z* 146.

Peak 18 with *λ*_max_ of 256.7 and 356.3 nm and a precursor ion [M−H]^−^ at *m*/*z* 477 was tentatively identified as quercetin-3-*O*-glucoronide. Characteristic fragments at *m*/*z* 301 and at *m*/*z* 300 [M-H-176]^−^ correspond to the quercetin aglycone and aglycone radical ion after the loss of the glucuronide molecule (176 Da). Other fragment ions at *m*/*z* 179 and at *m*/*z* 151 are characteristics of RDA cleavage of the aglycone quercetin [47].

#### 2.1.3. Quantification of the Main Marker Secondary Metabolites

The spectrophotometric quantification of the total phenolic content (TFC), total flavonoid content (TFC), and total condensed tannin content (TCT) detected in the PcAE and PcEE are depicted in Table 2. PcEE presented significantly higher amounts of TFC (415.2 ± 1.1 mg/g Dry Extract (DE)), TFC (229.1 ± 3.1 mg/g DE), and TCT (35.74 ± 0.03 mg/g DE) when compared to the values obtained for PcAE (212.1 ± 3.7 mg/g DE, 122.6 ± 3.2 mg/g DE and 17.43 ± 0.09 mg/g DE, respectively).

### 2.2. Effects of P. chevalieri on Posprandial Glycemia and Enzymatic Inhibition

The in vivo assay was conducted on 48 healthy CD1 mice. Animals were administered distilled water (2 control groups); 3 doses of PcEE (PcEE—D1 (40 mg/kg bw); PcEE—D2 (170 mg/kg bw), PcEE—D3 (300 kg/kg bw)) and acarbose (50 mg/kg bw) (antihyperglycemic drug) daily by gastric gavage for 14 days. On the 14th day, the animals were administered sucrose (3 g/kg) by gastric gavage 30 min after the administration of the extract doses, acarbose, or water (hyperglycemic control) with the exception of the control group assigned to be the normoglycemic control group.

#### 2.2.1. Modulation of Postprandial Glycemia

##### Clinical Signs

No relevant treatment-related clinical or behavioral irregularities were observed in the animals during the study period. A total of five animals (control, *n* = 2; PcEE-D2, *n* = 2; and PcEE-D3, *n* = 1) experienced acute mortality immediately following oral gavage. Given the rapid onset and clinical signs, these events were attributed to gavage-related administration errors. At the end of the experimental protocol, a macroscopic examination of the remaining animals (*n* = 43) revealed no lesions/changes in the vital organs (heart, kidneys, and liver) in comparison with the control group.

##### Body Weight and Food Intake Variation

The obtained data on the body weight and food intake of CD-1 mice administered vehicle (distilled water), PcEE, and acarbose in single doses for 12 days are shown in Table 3. Significant differences were observed in the percentage of body weight variation between the PcE-D2 group and the control group (*p* < 0.05), between the PcEE-D1 group and PcE-D2 group (*p* < 0.01), and between the PcEE-D1 and PcEE-D3 groups (*p* < 0.05). Regarding food consumption, no statistically significant differences were observed in the average daily food consumption for PcEE-treated groups and acarbose-treated groups when compared with the control group.

##### Oral Sucrose Tolerance Test

**Figure 3:**
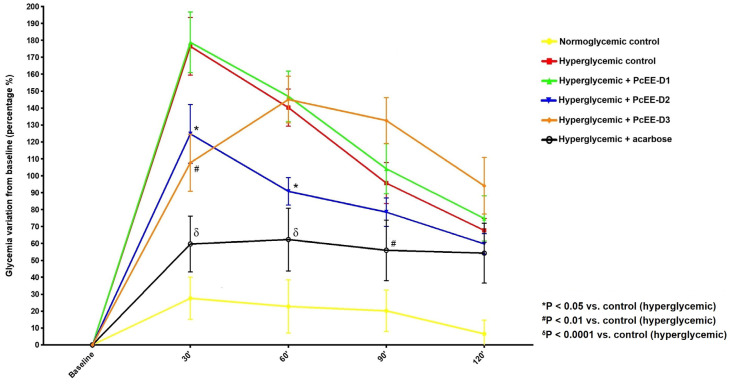
Blood glucose curves for the oral sucrose tolerance test (OSTT) in CD-1 mice treated with the *Periploca chevalieri* hydroethanolic extract (PcEE) or acarbose compared to the normoglycemic and hyperglycemic control groups. Doses: PcEE-D1, 40 mg/kg bw; PcEE-D2, 170 mg/kg bw; PcEE-D3, 300 mg/kg bw; and acarbose, 50 mg/kg bw. The blood samples were taken at 0, 30, 60, 90, and 120 min after the administration of sucrose (3 g/kg) by gavage. The data are presented as the means ± SEs (*n* = 6–8). Significance levels among different groups at *p* ≤ 0.05 (* *p* < 0.05 vs. control (hyperglycemic); ^#^ *p* < 0.01 vs. control (hyperglycemic); and ^δ^ *p* < 0.0001 vs. control (hyperglycemic).

The blood glucose curves obtained for the oral sucrose tolerance test (OSTT) in CD-1 mice gavaged with PcEE at different doses (40, 170, and 300 mg/kg bw) and acarbose (50 mg/kg bw) versus the normoglycemic and hyperglycemic control groups are depicted in Figure 3.

At all time points, the percentage of blood glucose remained unchanged in the normoglycemic control group.

The variations in the blood glucose percentage for all groups were compared to the variation in the blood glucose percentage of the hyperglycemic control group.

At 30 min post-sucrose administration, the hyperglycemic control group reached a blood glucose peak of approximately 175% compared to the basal glycemia measurement. The PcEE-D1 group presented similar behavior to the hyperglycemic control group, reaching the same blood glucose peak. The glycemia peak was significantly prevented in the PcEE-D2-, PcEE-D3-, and acarbose-treated groups compared to the hyperglycemic control group. For the PcEE-D2-and PcE-D3-treated groups, the post-prandial blood glucose levels were 54% (*p* < 0.05) and 69% (*p* < 0.01), respectively, which were significantly lower than the post-prandial increase in blood glucose levels observed for the hyperglycemic control group. With acarbose, the difference observed was even more pronounced and was 115% lower than the postprandial blood glucose peak observed in the hyperglycemic control group (*p* < 0.0001).

After 60 min, the percentage of blood glucose levels started to decrease in all groups, except for the PcEE-D3-treated group. The PcEE-D1-treated group continued to show the same behavior as the hyperglycemic control group. PcEE-D2- and acarbose-treated groups continued to show significantly lower percentages of blood glucose levels compared to the hyperglycemic control group; however, for the PcEE-D3-treated group, a significant increase in blood glycemia was observed.

At 90 and 120 min post-sucrose ingestion, a gradual decrease in glycemia was observed in all treated groups. At the end of the OSTT, the blood glucose percentage was comparable among all treated groups and the hyperglycemic control group.

Acarbose and PcEE-D2 doses demonstrated the potential to attenuate the postprandial glycemic peak following sucrose administration without reducing blood glucose levels below the baseline.

##### Biochemical Parameter Analyses

**Figure 4:**
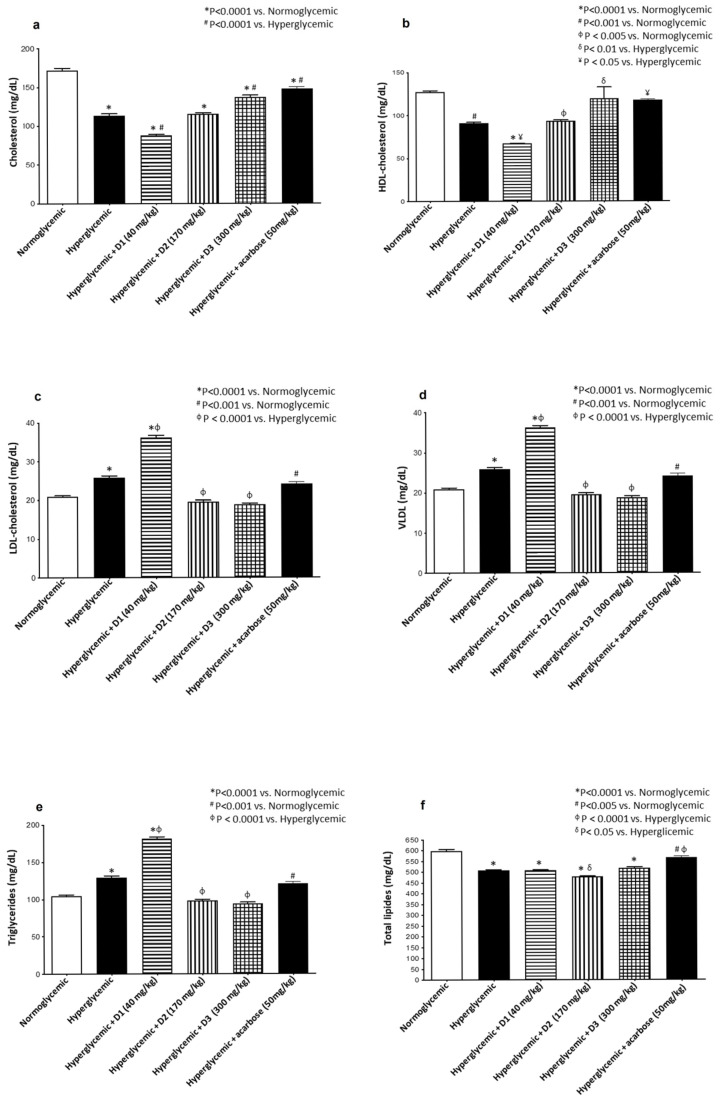
Lipid profile. Evaluation of mice gavaged with three different doses of the *P. chevalieri* aerial part extracts and acarbose to determine the levels of (**a**) cholesterol, (**b**) HDL, (**c**) LDL, (**d**) VLDL, (**e**) triglycerides, and (**f**) total lipids and a comparison with the normoglycemic and hyperglycemic control groups. Significance level among different groups at *p* ≤ 0.05.

The lipid profile, total cholesterol, HDL-cholesterol, LDL-cholesterol, very-low-density lipoprotein (VLDL)-cholesterol, triglycerides, and total lipids, were screened in the blood serum of all animals, and the results are presented in Figure 4.

The evaluation of the total cholesterol level (Figure 4a) indicated that all groups, including the hyperglycemic control group, presented significantly lower levels compared to the normoglycemic control. Regarding the HDL-cholesterol level (Figure 4b), the hyperglycemic control, PcEE-D1, and PcEE-D2 groups showed significantly lower levels than the normoglycemic control group. In contrast, all the PcEE-D3 and acarbose groups showed similar levels to the ones observed for the normoglycemic control group. Regarding LDL-cholesterol, VLDL-cholesterol, and triglyceride levels (Figure 4c–e), compared with the normoglycemic control group, the levels of these biomarkers were increased in the hyperglycemic control and PcEE-D1-treated groups, while in the PcEE-D2, PcEE-D3 and acarbose groups, no significant differences from the normoglycemic control group were observed.

The evaluation of the total lipid levels (Figure 4f) indicated that all groups, including the hyperglycemic control group, presented significantly lower levels compared to the normoglycemic control group.

#### 2.2.2. Inhibition of the α-Amylase, α-Glucosidase, and DPP4 Enzymes

The in vitro antihyperglycemic potential of PcAE and PcEE was studied by assessing their inhibitory activities against α-amylase and α-glucosidase, two of the main enzymes involved in the modulation of postprandial hyperglycemia, and DPP4, an enzyme responsible for the rapid inactivation of the incretins involved in the release of insulin from pancreatic β-cells. The inhibitory potential of the extracts was compared based on their IC_50_ values (Table 4). Acarbose and sitagliptin, two antidiabetic drugs used in the treatment of T2DM, were used as positive controls for the α-amylase and α-glucosidase inhibitory assays and DPP4 assay, respectively.

The results showed that both extracts inhibited all enzymes in a concentration-dependent manner. The IC_50_ values for the inhibition of α-amylase ranged from 19.1 ± 0.5 μg/mL for PcEE to 97.7 ± 1.5 μg/mL for PcAE. Compared to the positive control acarbose (11.4 ± 0.1 μg/mL), PcAE exhibited a low inhibitory activity while PcEE exhibited a moderate inhibitory activity. For the inhibition of the α-glucosidase enzyme, both extracts showed stronger inhibitory potential (PcAE IC_50_ value 17.7 ± 0.3 µg/mL and PcEE IC_50_ value 7.8 ± 0.2 µg/mL) than the positive control acarbose (350.3 ±15.3 µg/mL).

Regarding the ability of the extracts to inhibit the DPP4 enzyme, the obtained IC_50_ values for the extracts ranged from 1828.2 ± 3.6 to 1611.9 ± 9.3 μg/mL, whereas the IC_50_ of sitagliptin was 0.0154 ± 0.0003 μg/mL. In this assay, the IC_50_ values of the extracts and of sitagliptin were significantly different, with sitagliptin showing remarkably higher potency in the inhibition of the DPP4 enzyme compared to both extracts.

### 2.3. In Vitro Antioxidant Activity

The in vitro antioxidant potential of PcAE and PcEE was measured using four different assays: CUPRAC, FRAP, DPPH^•^ and O_2_^•−^ radical scavenging assays (Table 5).

The obtained CUPRAC values for all extracts differed significantly (*p* < 0.05), ranging from 438.9 ± 3.1 to 941.9 ± 38 mg/g. For the FRAP assay, a similar trend of the results was observed, with the highest antioxidant activity being displayed by PcEE (498.1 ± 1.5 mg/g, respectively) when compared with PcAE (238.1 ± 5.1 mg/g).

Regarding the ability to scavenge free radicals, all extracts showed DPPH and O2^•−^ radical scavenging activity in a concentration-dependent manner. PcEE, with an IC_50_ value of 23.9 ± 0.5 μg/mL, was the strongest DPPH radical scavenging extract, with a comparable IC_50_ value to that shown by ascorbic acid (17.3 ± μg/mL). The weakest ability to scavenge the DPPH radical was demonstrated by PcAE (49.0 ± 0.7 μg/mL).

For the ability to scavenge the O_2_^•−^ radicals, both PcAE and PcEE presented lower IC_50_ values (15.7 ± 1.4 and 7.0 ± 1.0 μg/mL, respectively) compared to the positive control (gallic acid, 16.0 ± 3.1 μg/mL).

### 2.4. Evaluation of the Antiglycation Capacity

The potential of PcAE and PcEE to inhibit protein glycation was assessed through the inhibition of bovine serum albumin (BSA) glycation. The inhibition of AGE formation in the last stages glycation in vitro was evaluated in systems consisting of BSA and the sugars fructose (FRU) glucose (GLU). The inhibition of AGE formation by the extracts was compared based on their IC_50_ values, and the antiglycation drug aminoguanidine was used as positive control in both systems (Table 6).

The results obtained showed that both PcAE and PcEE inhibited the glycation of BSA in a dose-dependent manner in the tested systems, and both extracts showed a significantly higher antiglycation capacity than the antiglycation drug aminoguanidine.

PcEE presented lower IC_50_ values in both the BSA-FRU (11.0 ± 0.2 µg/mL) and BSA Glu (19.7 ± 0.1 µg/mL) systems compared to the IC_50_ values of PcAA (23.0 ± 0.5 µg/mL, 24.3 ± 0.1 µg/mL, respectively). Nevertheless, in all systems, both extracts presented significantly lower IC_50_ values than aminoguanidine.

## 3. Discussion

*Periploca chevalieri* is a medicinal plant endemic to the Cabo Verde Archipelago that is traditionally used to manage diabetes. This study aimed to identify the main marker secondary metabolites present in *P. chevalieri* aerial parts, and to evaluate their potential to modulate postprandial glycemia. Additionally, their capacity to manage oxidative stress and protein glycation was also evaluated.

Two types of extracts were prepared, an aqueous extract (PcAE) prepared by an infusion simulating the traditional preparation used in Cabo Verde, and a 70% hydroethanolic extract (PcEE) obtained by maceration, a common method of preparation used to obtain total extracts from plant raw materials.

Both extracts displayed qualitatively similar chemical profiles; however, PcEE was superior in terms of the total phenolic contents, flavonoid contents, and condensed tannin contents compared to PcAE, as the hydroethanolic solvent (30:70 v/v) is normally more efficient in extracting phenolic compounds than hot water.

The chemical screening of *P. chevalieri* aerial part extracts allowed the tentative identification of phenolic acids, flavan-3-ols, proanthocyanidins, and flavonols through LC/UV-DAD-ESI/MS/MS spectral data, data from co-chromatography with authentic standards, and the relevant literature. Twenty compounds, namely, quinic acid (1), gallocatechin (2), B-type proanthocyanidin dimers (3, 4, 7, and 8), B-type proanthocyanidin trimers (5 and 9), 3-*O*-caffeoyilquinic acid (6), epigallocatechin (10), catechin (11), 4-*O*-caffeoyilquinic acid (12), 5-*O*-caffeoyilquinic acid (13), epicatechin (14), quercetin glycosides (15 and 16), quercetin 3-*O*-rutinoside (17), quercetin 3-*O*-glucoronide (18), quercetin 3-*O*-rutinoside (19), and 1,4-dicaffeoyilquinic acid were identified as major marker compounds of *P. chevalieri* extracts.

Based on the preliminary quantification results, PcEE was selected for the OSTT.

Oral glucose and sucrose tolerance tests are standard procedures employed to evaluate the effectiveness of substances with the potential to modulate postprandial glycemia after the oral ingestion of a standard amount of glucose or sucrose [48].

The tolerance test was performed with sucrose to evaluate the potential of PcEE to influence sucrose digestion and glucose absorption.

Since sucrose must be hydrolyzed into glucose and fructose by α-glucosidase in the small intestine before absorption, changes in postprandial blood glucose levels reflect α-glucosidase activity. However, if α-glucosidase is inhibited, sucrose breakdown is reduced, leading to a slower or lower rise in blood glucose levels than the hyperglycemic control [49]. The OSTT results showed that experimental groups treated with the *P. chevalieri* extract (170 and 300 mg/kg bw) and acarbose (50 mg/kg bw) were able to significantly prevent postprandial hyperglycemia in the first 30 min after sucrose overload compared to hyperglycemic control group, indicating a potential antihyperglycemic effect.

After 60 min, the glycemia-modulating effect remained evident in the PcEE-D2-treated group (170 mg/kg bw); however, for the PcEE-D3 group (300 mg/kg bw), an unexpected outcome was observed. Instead of an antihyperglycemic effect, a hyperglycemic response was observed. This contradictory result could be justified by the complex phytochemical composition of the extract. Phytochemicals with potential antagonistic interactions may produce non-linear dose–response relationships, including biphasic patterns in complex extracts [50]. According to Calabrese et al. and Siwak et al., polyphenols such as quercetin, rutin, catechin, and epigallocatechin gallate can exhibit stimulatory effects at lower doses and diminished or even opposite activity at higher concentrations [51,52]. These results highlight the importance of dose optimization and support the need for further mechanistic studies to elucidate the pharmacodynamics of the extract and its constituents.

Acarbose, used as a positive control, was more effective than all *P. chevalieri* extract doses. Nevertheless, the attenuation of the glycemic peak observed in the PcEE-treated group (170 mg/kg bw) compared to the hyperglycemic control group supports the hypothesis that the extract interferes with the carbohydrate hydrolyzation mechanism mediated by α-glucosidase. This mechanism is consistent with that of standard α-glucosidase inhibitors, such as acarbose, and further reinforces *P. chevalieri*’s potential as a postprandial glucose modulator.

Dyslipidemia, characterized by elevated levels of cholesterol, LDL, and triglycerides and reduced levels of HDL, is commonly associated with diabetes [53]. In this study, treatment with PcEE-D2, PcEE-D3, and acarbose resulted in relatively stable lipid profiles when compared to the normoglycemic group. However, compared to the hyperglycemic control group, these treatments produced significant improvements in dyslipidemia markers, including reductions in LDL, VLDL, and triglyceride levels, along with a significant increase in HDL levels. In contrast, the PcEE-D1-treated group showed elevated LDL, VLDL, and triglyceride levels, suggesting a subtherapeutic and potentially unfavorable metabolic effect of this lower dose. These findings highlight the importance of dose optimization and support the use of the mid-range dose (PcEE-D2) as the most effective and metabolically balanced option.

The lipid-modulating effects observed with PcEE-D2 and PcEE-D3 align with previous studies reporting that medicinal plants can improve lipid profiles by lowering total cholesterol, triglyceride, and LDL levels while increasing HDL levels [53]. Although the results indicate a hypolipidemic effect of PcEE (D2 and D3), further investigation is needed to fully characterize its impact on lipid metabolism.

Prior to the OSTT, animals were fed a standard diet and received a daily oral administration of either the vehicle (control group), PcEE extract, or acarbose for 13 days. Among the groups, only the animals treated with PcE-D2 (170 mg/kg bw) showed a significant change in body weight compared to the control group. Interestingly, this was also the group that most effectively attenuated the postprandial glycemic peak following sucrose administration. These findings suggest that *P. chevalieri* may have potential to promote weight management through glycemic regulation. However, further studies in specific models of obesity or T2DM (e.g., db/db mice) are needed to confirm the potential effects.

To date, no previous work has investigated the antihyperglycemic potential of *P. chevalieri*. However, other species within the *Periploca* genus have demonstrated antidiabetic activity. Oral administration of a 500 mg/kg ethanolic extract of *P. angustifolia* significantly reduced blood glucose levels in alloxan-induced diabetic rats [54]. Similarly, a *P. aphylla* methanolic extract and various subfractions (n-hexane, chloroform, ethyl acetate, and n-butanol) at doses of 200 and 400 mg/kg [55] showed significant antihyperglycemic effects on both glucose-overloaded normoglycemic rats and streptozotocin-induced diabetic models.

Both studies attributed the observed antidiabetic potential to the chemical compositions. Chemical characterization of the *P. angustifolia* extract identified compounds like coumarin, resorcinol, isorhamnetin, quercetin, and naphthalene, while the *P. aphylla* extracts’ chemical profiles identified the presence of rutin, catechin, caffeic acid, and myricetin. Rutin and catechin, two of the main compounds identified in *P. aphylla*, are some of the main marker compounds identified in *P. chevalieri* extracts, together with several quercetin glycosides. The observed phytochemical profile and pharmacological potential reinforce the therapeutic relevance of the *Periploca* genus in the management of T2DM.

The effective management of postprandial hyperglycemia is a key factor in the management of T2DM. Thus, preventing an excessive postprandial blood glucose rise or maintaining a blood glucose limit within the normal range is still a very effective therapeutic strategy for the management of T2DM [4]. The enzymes *α*-amylase and *α*-glucosidase have been exploited as drug targets for preventing postprandial hyperglycemia in T2DM. *α*-Amylase, occurring in the saliva and pancreas, is responsible for the cleavage of carbohydrates’ *α-*(1,4)-*D*-glycosidic bonds to produce oligosaccharides, which are further cleaved to monosaccharide glucose by *α*-glucosidase located in the brush-border surface membrane of the intestinal cells [10,56].

The potential of *P. chevalieri* aerial part extracts to inhibit both enzymes was therefore investigated as a probable mechanism of action related to the observed in vivo antihyperglycemic effect of PcEE. The obtained results showed that, indeed, *P. chevalieri* extracts were able to inhibit both *α*-amylase and *α*-glucosidase in a dose-dependent manner. Both PcAE and PcEE exhibited strong *α*-glucosidase enzyme inhibition and moderate (PcEE) to low (PcAE) inhibition of the *α*-amylase enzyme when compared to acarbose.

According to molecular docking studies, compounds such as syringic acid, hesperetin, narenginin, ferulic acid, curcumin, cyanidin, daidzein, epicatechin, eridyctiol, pinoresinol, quercetin, and resveratrol showed strong abilities to inhibit the *α*-glucosidase enzyme. However, compounds such as pelargonidin, hesperetin, kaempferol, silibinin, and catechin showed strong inhibition of the *α*-amylase enzyme [57]. Consequently, the fact that PcAE and PcEE exhibited higher amounts of flavonoids, like quercetin derivatives, and lower amount of tannins, like catechin derivatives, may be the reason why both extracts have shown better activity against *α*-glucosidase compared to *α*-amylase.

Available *α*-glucosidase inhibitors, such as acarbose, miglitol, and voglibose, are useful in lowering postprandial hyperglycemia, however they can have gastrointestinal adverse effects like flatulence, diarrhea, and stomach pain. Although these symptoms are typically not severe, they are the most frequent causes of reduced compliance and treatment discontinuation [8].

According to Rasouli et al. [57], the gastrointestinal adverse effects related to *α*-glucosidase inhibitors are related to their potent and non-selective inhibition of pancreatic *α*-amylase. Therefore, the discovery of new inhibitor drugs with strong inhibitory action against *α*-glucosidase and moderate to weak inhibitory properties for pancreatic *α*-amylase is of great importance. The obtained results for the inhibition of both enzymes showed that there may be a decreased risk of gastrointestinal adverse effects linked to *P. chevalieri* extracts due to the significant *α*-glucosidase inhibitory actions and the moderate to weak *α*-amylase inhibitory effects of both extracts.

Further insights into other mechanisms of action related to the observed in vivo antihyperglycemic effect of PcEE were evaluated by determining the potential of *P. chevalieri* extracts to enhance insulin secretion by inhibiting the DPP4 enzyme. DPP4 is a serine protease ectoenzyme that is present in the gastrointestinal tract, kidneys, and endothelial layer of blood vessels and contributes to the regulation of various physiological processes, namely, blood glucose homeostasis [58,59].

DPP4 inhibition has become a novel target for the incretin system-focused treatment of T2DM [60]. DPP4 accelerates the degradation of the incretin hormones glucagon-like peptide-1 (GLP-1) and glucose-dependent insulinotropic polypeptide (GIP) [58]. The inhibition of DPP4, therefore, prevents the degradation of these hormones, enhancing glucose-stimulated insulin secretion (incretin action).

PcAE and PcEE extracts inhibited DPP4 up to 75% and 82% at maximum concentrations of 3.0 and 2.5 mg/mL, respectively. Although considerably less effective than the pure preparation of sitagliptin, these results suggest that the inhibition of the DPP4 enzyme may contribute to the antihyperglycemic mechanism of action of *P. chevalieri*. In fact, some of the main marker compounds (e.g., catechin, rutin, and quercetin glycosides) identified in both extracts have been shown to inhibit the DPP4 enzyme [60,61]. According to [61], catechin, epicatechin, isoquercitrin, quercetin, and rutin are potent inhibitors of DPP4, exhibiting low effective concentrations (isoquercitrin and quercetin inhibited at 25–50 μM whereas rutin was particularly effective with maximal inhibition of 45% and an IC_25_ value of 306 μM).

A persistent hyperglycemic state leads to enhanced oxidative stress and non-enzymatic glycation of proteins, which are associated with secondary complications such as diabetic neuropathy, nephropathy, retinopathy, and cardiovascular diseases in patients with T2DM [62]. Glycation is a spontaneous process involving a sequence of non-enzymatic reactions between reducing sugars and amino groups found in proteins, lipids, and nucleic acids to form a Schiff base, which is then followed by an Amadori rearrangement and oxidative modifications (glycoxidation) that result in the production of AGEs [63,64].

During hyperglycemia, the synthesis of Amadori products and AGEs accelerates, and ROS levels rise dramatically. Glycation stress is intrinsically linked to oxidative stress because AGE production increases reactive oxygen species formation and impairs antioxidant systems; on the other hand, AGE formation is triggered by oxidative circumstances [64].

In this sense, the pharmacological prevention of protein glycation and oxidative stress has become an attractive means of preventing diabetic micro- and macrovascular complications.

According to Ortega-Castro et. al., the mechanisms of action of AGE inhibitors are the scavenging of carbonyl and radical species and the inhibition of the oxidation of Amadori compounds by chelating metal ions, such as Cu^2+^ and Fe^3+^, which catalyze the Amadori compounds’ autooxidation [65].

Aminoguanidine, which was used as positive control in the antiglycation assay, is effective against AGE formation via its antioxidant action [66]. It forms complexes with metals that slow down glycation and subsequent AGE formation by scavenging the metal cations Cu^2+^ and Fe^3+^ [67,68,69].

In this study, both PcAE and PcEE demonstrated strong antiglycation activity, significantly exceeding that of the antiglycation drug aminoguanidine. Both extracts also exhibited a high antioxidant capacity. The observed reducing capacity of the extracts, as shown in CUPRAC and FRAP assays, along with their ability to scavenge the superoxide anion radical suggests that the antioxidant and antiglycation potential of *P. chevalieri* is strongly connected to its chemical composition.

Phenolic compounds such as chlorogenic acid, quercetin glycosides (rutin and hyperoside), catechin, and proanthocyanidins, which are the main marker compounds of *P. chevalieri*, are widely recognized for their antioxidant and antiglycation properties [22,70] Several studies have demonstrated that quercetin and rutin inhibit AGE formation more effectively than reference drug aminoguanidine. Quercetin inhibits AGE formation by scavenging reactive carbonyl compounds, chelating metal ions, trapping methylglyoxal, and trapping reactive oxygen species [71,72]. Rutin inhibits AGE formation by scavenging reactive carbonyl compounds and preventing protein oxidation and protein crosslink formation [73,74].

Chlorogenic acid has been reported to suppress AGE formation and protein cross-linking, while also acting as a strong antioxidant agent [70,75]. Catechins and proanthocyanidins, especially their oligomeric forms, exhibit potent antiglycation effects by trapping reactive carbonyl species like methylglyoxal [70].

Additionally, molecular docking studies support these findings by demonstrating high binding affinities of these compounds to AGE receptors and oxidation targets [76,77]. For example, chlorogenic acid shows strong interactions with aldose reductase, an enzyme involved in ROS generation and glycation pathways [78].

Together, these results highlight the therapeutic relevance of *P. chevalieri* as a polyphenol-rich extract capable of interfering with oxidative and glycation pathways.

## 4. Materials and Methods

### 4.1. Chemicals, Reference Items, and Reagents

β-Nicotinamide adenine dinucleotide (NADH), acarbose, and gallic acid and were purchased from AlfaAesar (Karlsruhe, Germany). Ethanol was purchased from Carlo Erba Reagents (Val-de-Reuil, France). Neochlorogenic acid (3-*O*-caffeoylquinic acid), chlorogenic acid (5-*O*-caffeoylquinic acid), (+)-catechin, (−)-epicatechin, quercetin-3-*O*-rutinoside (rutin), and quercetin-3-*O*-galactoside (hyperoside) were obtained from Extrasynthese (Genay, France). Di-potassium hydrogen phosphate anhydrous and sodium chloride were purchased from Fluka (Seelze, Germany). Folin–Ciocalteu reagent, iron (III) chloride hexahydrate, methanol, sodium carbonate, sodium hydroxide, sodium nitrite, and sitagliptin were purchased from Merck (Darmstadt, Germany). Ascorbic acid, disodium hydrogen phosphate dihydrate, hydrochloric acid, sodium acetate trihydrate, and sodium dihydrogen phosphate monohydrate were purchased from Panreac (Barcelona, Spain). Ammonium acetate, copper (II) chloride dihydrate, and iron sulfate heptahydrate were purchased from Riedel-de Haën (Seelze, Germany). *α*-Amylase from porcine pancreas, *α*-glucosidase from Sacharomyces cerevisiae, 2,2-diphenyl-1-picrylhydrazyl (DPPH), 2,4,6-tris(2-pyridyl)-s-triazine (TPTZ), 3,5-dinitrosalicylic acid, 4-nitrophenyl-*α*-D-glucopyranoside (NPG), 4-nitroblue tetrazolium chloride (NBT), aluminum chloride, bovine serum albumin, D-(+)-glucose monohydrate, the DPP4 inhibitor screening kit (MAK203), neocuproine, soluble potato starch, and phenazine methosulfate (PMS) were purchased from Sigma-Aldrich (St. Louis, MO, USA). Formic acid (99–100%) was from VWR (Radnor, PA, USA). Aminoguanidine was obtained from Tokyo Chemical Industry, (Tokio, Japan). Ultra-pure water from a Milli-Q water purification system (Millipore, Molsheim, France) was used to prepare all the solutions and dilutions.

### 4.2. Plant Material Collection and Preparation of Extracts

*P. chevalieri* aerial parts were collected from the Natural Park of Serra Malagueta, Cabo Verde, Santiago Island. Botanical identification was carried out by Samuel Gomes, and a voucher specimen (042016KL) was preserved in the herbarium of INIDA, Santiago, Cabo Verde. The collected plant material was air-dried in the shade and kept in the dark until use.

*P. chevalieri* aqueous extract (PcAE) was prepared by adding 1 L of boiling ultrapure water (95 °C) to 15 g of coarsely chopped dried plant material and allowing it to stand and infuse for 5 min. After cooling, the obtained extract was filtered, frozen to −20 °C ± 1 °C, freeze-dried (Heto, LyoLab 3000, Termo Fisher Scientific, Leicestershire, UK), and stored in the dark at room temperature. To prepare the *P. chevalieri* hydroethanolic extract (PcEE), dried and pulverized aerial parts were extracted exhaustively (25 °C, 72 h) with 70% ethanol in water at a ratio of 1:10 (1 part plant powder to 10 parts solvent). The obtained extract was then filtered and evaporated under reduced pressure at a temperature of 40 °C ± 1 °C, frozen at −20 °C ± 1 °C, freeze-dried (Heto, LyoLab 3000, Termo Fisher Scientific, Leicestershire, UK), and finally stored in the dark at room temperature.

### 4.3. Chromatographic Conditions

The HPLC/UV-DAD analysis was performed on a Liquid Chromatograph Waters Alliance 2690 Separations Module (Waters Corporation, Milford, MA, USA) coupled with a diode array detector (Waters 966 PDA; Waters Corporation, Milford, MA, USA). The column used was a Purospher STAR RP-18 endcapped column (4 × 250 mm, 5 μm), with a pre-column (4 × 250 mm, 5 μm) from Merck (Darmstadt, Germany). The mobile phase consisted of a linear gradient of water + 0.1% formic acid as solvent A and methanol as solvent B (v/v: 0 min, 95% A, 5% B; 8 min, 89% A, 11% B; 40 min, 71% A, 29% B; and 65 min, 0% A, 100% B. The injection volume was 10 µL and the analysis was performed at a flow rate of 1 mL/min and column temperature of 25 ± 5 °C.

The chromatograms were recorded at maximum absorbance of each band (MaxPlot) between 210 and 450 nm, and Waters Millennium^®^ 32 V 3.0 Chromatography Software (Waters Corporation, Milford, MA, USA) was used for data analysis.

The HPLC-ESI/MS/MS analysis was performed on Liquid Chromatograph Waters Alliance 2695 Separations Module (Waters Corporation, Milford, MA, USA) HPLC, with a photodiode array detector and an autosampler (Waters PDA 2996) in tandem with a triple quadrupole mass spectrometer (Micromass^®^ Quatro MicroTM API, Waters^®^, Drinagh, Ireland) with an electrospray ionization source (ESI) functioning in negative mode. The column used was a LiChrospher 100 RP-18 (5 µm) 250 × 4 mm column with a pre-column (Merck, Darmstadt, Germany). The mobile phase consisted of a linear gradient of water + 0.1% formic acid as solvent A and methanol as solvent B (v/vV): 0 min, 95% A, 5% B; 20 min, 80% A, 20% B; 60 min, 50% A, 50% B; and 90 min, 0% A, 100% B. The injection volume was 20 µL, the flow rate used was 0.3 µL/min, and the column temperature was 25 ± 5 °C.

The obtained data were analyzed using MassLynx™ V4.1 software (Waters^®^, Drinagh, Ireland).

Samples were prepared in water (PcAE) or methanol 70% (PcEE) at concentrations of 10 mg/mL for the LC/UV-DAD analysis and in 50% methanol at concentrations of 30 mg/mL for the LC-ESI/MS/MS analysis. The standards were prepared at concentrations of 1 mg/mL.

### 4.4. Quantification of the Main Classes of Secondary Metabolites

Total phenolic and flavonoid contents were estimated according the methods previously described by Lima et al., 2023 [79], and the condensed tannin content was estimated according to the method described by Sabiha et al., 2024 [80].

The total phenolic content was quantified by the Folin–Ciocalteu method. The absorbance was spectrophotometrically recorded at 765 nm (SPEKOL 1500, Analytik Jena, Jena, Germany). The total phenolic amount was calculated using a gallic acid calibration curve (concentration range: 100–700 μg/mL; linear regression equation: y = 0.0896x + 0.0281; R^2^ = 0.9986) and the results are expressed as mg of gallic acid equivalents per g of dry extract (mg GAEs/g).

For the total flavonoid content estimation, the absorbance was spectrophotometrically recorded at 510 nm (SPEKOL 1500, Analytik Jena, Jena, Germany). The flavonoid amount was calculated using a catechin calibration curve (concentration range: 18–290 μg/mL; linear regression equation: y = 0.0032x + 0.0029, R^2^ = 0.9991), and the results are expressed as mg of catechin equivalents per g dry extract (mg CEs/g).

For the condensed tannin content estimation, the absorbance was spectrophotometrically recorded at 550 nm (SPEKOL 1500, Analytik Jena, Germany). The condensed tannin amount was calculated using a cyanidin chloride calibration curve (concentration range: 50–300 μg/mL; linear regression equation: y = 0.003x + 0.0093, R^2^ = 0.9994), and the results are expressed as mg of cyanidin chloride equivalents per g dry extract (mg CEs/g DE).

### 4.5. In Vivo Antihyperglycemic Potential

#### 4.5.1. Animals

Male CD-1 mice (5–10 weeks old, 28–40 g) were obtained from Institute of Hygiene and Tropical Medicine (IHMT), Universidade NOVA de Lisboa. The acclimatization process and feeding conditions were consistent with the work of Malú et al., 2023 [81]. The animals were kept for seven days in rooms with a temperature of 22 ± 3 °C, relative humidity of 40–60%, and a 12 h light/dark cycle at Animal Facility of the Faculty of Pharmacy, Universidade de Lisboa. The animals were maintained in cages and had free access to water and food (standard laboratory chow (4RF21 GLP; Mucedola srl, Milan, Italy)).

All procedures were conducted in agreement with the animal welfare organization of the Faculty of Pharmacy, Universidade de Lisboa (protocol CEEE-002/16 approved by the Ethics Committee for Animal Experiments (CEEA) in 26 February 2016.), represented by the expert national authority “Direção Geral de Alimentação e Veterinária” (DGAV) according to the EU Directive (2010/63/UE) and the Portuguese laws (DR 113/2013, 2880/2015, and 260/2016). Additionally, all the experiments were performed according to the ARRIVE Guidelines for Reporting Animal Research.

#### 4.5.2. Experimental Protocol

The assay was conducted by following the protocol of Grácio et al., 2021 [82], with some modifications. A total of 48 CD1 mice were allocated into experimental groups and received daily oral treatments for 14 consecutive days. The control group (*n* = 16 animals) was treated with the vehicle (distilled water), while the reference group received acarbose at a dose of 50 mg/kg bw (*n* = 8). The experimental groups were treated with the PcEE at the following doses: PcEE-D1 (40 mg/kg bw) (*n* = 8), PcEE-D2 (170 mg/kg bw) (*n* = 8), and PcEE-D3 (300 mg/kg bw) (*n* = 8), all dissolved in the vehicle. Treatments were administered via oral gavage (10 mL/kg bw).

On the 14th day (OSTT day), the control group (*n* = 16) was subdivided into two subgroups: the normoglycemic control group (*n* = 8) and the hyperglycemic control group (*n* = 8).

##### Repeated Administration (14 Days)

Animals were administered treatments daily by gastric gavage for 14 days with the last administration being the final day, 30 min prior to basal glycemic measurement and hyperglycemic induction.

Animals were randomly allocated to six experimental groups:

- Normoglycemic group—animals were treated orally with the vehicle (distilled water) for 14 days, and on the last day, the vehicle was administered instead of sucrose;
- Hyperglycemic group—animals were treated with the vehicle for 14 days, and on the last day, a solution of sucrose (3 g/kg) was administered by gastric gavage;
- PcE-D1 group—animals were treated orally with PcEE (40 mg/kg BW) for 14 days, and on the last day, a solution of sucrose (3 g/kg) was administered by gastric gavage;
- PcE-D2 group—animals were treated orally with PcEE (170 mg/kg BW) for 14 days, and on the last day, a solution of sucrose (3 g/kg) was administered by gastric gavage;
- PcE-D3 group—animals were treated orally with PcEE (300 mg/kg BW) for 14 days, and on the last day, a solution of sucrose (3 g/kg) was administered by gastric gavage;
- Acarbose group—animals were treated orally with acarbose (50 mg/kg BW) for 14 days, and on the last day, a solution of sucrose (3 g/kg) was administered by gastric gavage.

##### Experimental Oral Sucrose Tolerance Test (OSTT)

On day 14, the animals that had fasted overnight received a single dose of sucrose (3 g/kg) 30 min after sample administration to all groups, except for the normoglycemic control group, which received water. Glycemia measurements in blood collected by tail vein puncture were made at times (*t*) *t*0 (immediately before the administration of the sucrose solution for basal values) and at *t*30, *t*60, *t*90 and *t*120 min after sample administration. The measurements were determined with a portable glucometer (Contour next, Basel, Switzerland).

#### 4.5.3. Blood Collection, Necropsy, and Biochemical Analysis

At the end of the experiment, all animals were euthanized by an anesthetic overdose with a mixture of ketamine–xylazine (100 mg/kg:10 mg/kg), blood samples were collected via cardiac puncture, and organs were harvested for further histopathological studies, if justified. The necropsy also included a thoracic and abdominal macroscopic examination. Serum samples obtained from animals’ blood samples centrifuged at 4000 rpm (JP Selecta, Barcelona, Spain) for 15 min were stored at 20 ± 1 °C until analysis.

Serum levels of lipid biomarkers (total cholesterol, high-density lipoprotein (HDL-cholesterol), low-density lipoprotein (LDL-cholesterol), very-low-density lipoprotein (VLDL-cholesterol), triglycerides, and total lipids) were evaluated in the laboratory of Clinical Analysis Joaquim Chaves certified under the Quality management systems requirements (EN ISO 9001:2015 Pt, Instituto Português da Qualidade) ISO 90001-2015 standard. The analytical determinations were made according to the protocols described by Grácio et al., 2021 [82].

### 4.6. In Vitro Antihyperglycemc Potential

#### 4.6.1. α-Amylase Enzyme Inhibition Assay

The inhibition of the *α*-amylase enzyme was determined according to the method described by Romeiras et al., 2023 [24]. Briefly, reaction mixtures containing 100 µL of Type VI-B porcine pancreatic *α*-amylase (0.5 mg/mL in 100 mM sodium phosphate buffer containing 6.7 mM sodium chloride, pH 6.7) and 100 µL of extracts in appropriate dilutions were preincubated in test tubes at 37 °C for 10 min. Then, 100 µL of 1% starch (*w*/*v*, previously suspended in 100 mM sodium phosphate buffer containing 6.7 mM sodium chloride, pH 6.7, and boiled for 10 min) was added. After 10 min at 37 °C, 200 µL of DNS reagent (consisting of 20 mL of 96 mM DNS, 8 mL of 5.315 M sodium potassium tartrate tetrahydrate in 2 M NaOH, and 12 mL of distilled water) was added. The reaction mixtures were heated at 100 °C for 15 min to stop the reaction then cooled to room temperature and diluted with 2 mL of distilled water. The intensity of the reddish color was spectrophotometrically measured at 520 nm (SPEKOL 1500, Analytik Jena, Jena, Germany). The reaction mixture without the extracts was used as negative control and the reaction mixtures without *α*-amylase were used as the sample blanks. Increasing concentrations of acarbose (10–125 µg/mL) were used as positive controls. The results were expressed as the concentration in reaction mixture that reduced the enzyme activity by 50% (IC_50_). The enzyme inhibitory rate was calculated according to the following formula:

$$Inhibition\;(\%)=[(NC\;Abs-\left(S\;Abs-SB\;Abs\right))⁄NC\;Abs]\times 100$$

where NC Abs is the absorbance of the negative control, S Abs is the absorbance of the sample, and SB Abs is the absorbance of the sample blank.

#### 4.6.2. α-Glucosidase Enzyme Inhibition Assay

The inhibition of the *α*-glucosidase enzyme was determined according to the colorimetric method described by Rouzbehan et al., 2017 [83], with some modifications. Reaction mixtures containing 5 µL of *α*-glucosidase (6.25 U/mL in phosphate buffer (pH 6.9, 0.1 M)), 125 µL of phosphate buffer (pH 6.9, 0.1 M), and 20 µL of the plant extracts at different concentrations were prepared in a 96-well microplate (Greiner Bio-One, Rainbach im Mühlkreis, Austria) and incubated for 15 min at 37 °C. The reaction was started by adding 20 µL of the substrate solution (p-nitrophenyl-α-D-glucopyranoside, 2.75 mM in phosphate buffer (pH 6.9, 0.1 M)) and the plates were incubated for an additional 15 min at 37 °C. The reaction was stopped by the addition of 80 µL of 0.2 M Na_2_CO_3_. The absorbance of the wells was measured in a microplate reader (FLUOstar^®^ Omega Plate Reader, V5.70, BMG 264 Labtech, Ortenberg, Germany) at 405 nm. The reaction mixture without the extracts was used as negative control and reaction mixtures without *α*-glucosidase were used as sample blanks. Increasing concentrations of acarbose (1–12 mg/mL) were used as positive controls. The results were expressed as the concentration in the reaction mixture that reduced the enzyme activity by 50% (IC_50_). The enzyme inhibitory rate was calculated as follows:

$$Inhibition\;(\%)=[(NC\;Abs-\left(S\;Abs-SB\;Abs\right))⁄NC\;Abs]\times 100$$

where NC Abs is the absorbance of the negative control, S Abs is the absorbance of the sample, and SB Abs is the absorbance of the sample blank.

#### 4.6.3. DPP4 Enzyme Inhibition Assay

A DPP4 inhibitor screening kit (Sigma-Aldrich, MAK203) was used to evaluate the DPP4 inhibitory activity of the test samples based on a fluorescence assay method. Briefly, 25 μL of samples at different concentrations dissolved in water was pipetted into a 96-well black plate (clear flat-bottom plate (Greiner Bio-One, Rainbach im Mühlkreis, Austria) followed by the addition of 50 μL of diluted DPP4 enzyme solution (1 μL enzyme in 49 μL of assay buffer). After a 10 min incubation at 37 °C, 25 μL of the diluted substrate solution (2 μL of substrate in 23 μL of assay buffer) was added to the reaction mixture. Then the plate was shaken, and the fluorescence was monitored in kinetic mode for 20 min (readings every minute) at 37 °C at an excitation wavelength of 360 nm and an emission wavelength 460 nm in a microplate reader (FLUOstar^®^ Omega Plate Reader, V5.70, BMG 264 Labtech, Ortenberg, Germany). An enzyme control was prepared using DPP4 assay buffer in place of the sample inhibitor and for the positive control, the sample was replaced by the sitagliptin standard. Each extract concentration generated a fluorescence curve as a function of time. The slope (Δfluorescence/min) of the linear domain of these curves was used to determine the inhibition, as a percentage, of the enzyme activity according to the following formulas:

Slope=[(FLU2−FLU1)⁄(T2−T1)]=∆FLU/minute

where FLU 2 is the fluorescence at time 2, FLU 1 is the fluorescence at time 1, T2 is time 2, and T1 is time 1.

Inhibition (%)=[(SlopeEC−Slope SI)⁄(Slope Ec)]×100

where SlopeEC is the slope of the enzyme control (negative control) and SlopeSI is the slope of the sample inhibitor.

### 4.7. Antioxidant Capacity

The antioxidant activity was evaluated by spectrophotometric techniques and CUPRAC (cupric reducing antioxidant capacity), FRAP (ferric reducing antioxidant power), DPPH (2,2-diphenyl-1-picrylhydrazyl), and O2^•−^ (superoxide anion) radical scavenging assays.

#### 4.7.1. CUPRAC Assay

The assay was performed according to the method described by Lima et al., 2023 [79]. In a test tube, 1 mL of the following solutions were mixed: copper (II) chloride dihydrate (10 mM), ammonium acetate (1 M), and neocuproine in ethanol (7.5 mM). Then, 300 μL of the extracts at appropriate dilutions was added to the previous mixture and water up to a final volume of 4.1 mL. After a 1 h incubation at room temperature, the absorbance was spectrophotometrically recorded at 450 nm (SPEKOL 1500, Analytik Jena, Jena, Germany). A calibration curve of ascorbic acid (concentration range: 2–45 µg/mL; linear regression equation: Y = 0.025x + 0.057, R2 = 0.9966) was used and the results were expressed as mg of ascorbic acid equivalents per g of dry extract (mg AAEs/g).

#### 4.7.2. FRAP Assay

The assay was performed according to the procedure described by Lima et al., 2019 [84]. In test tubes, 100 µL of extracts at appropriate dilutions was mixed with 3 mL of freshly prepared and pre-warmed (37 °C) FRAP reagent (0.25 M sodium acetate buffer, pH 3.6, 10 mM TPTZ solution in 40 mM HCl, and 20 mM FeCl_3_.6H_2_O at a ratio of 10:1:1). After 4 min of incubation at 37 °C, the absorbance of the mixture was spectrophotometrically recorded at 593 nm (SPEKOL 1500, Analytik Jena, Jena, Germany). A calibration curve of ascorbic acid (concentration range: 25–125 µg/mL; linear regression equation: Y = 0.2692x + 0.0871, R^2^ = 0.9941) was used and the results are expressed as mg of ascorbic acid equivalents per g of dry extract (mg AAEs/g).

#### 4.7.3. DPPH Radical Scavenging Assay

The DPPH radical scavenging assay was performed according to the method described by Miceli et al., 2009 [85]. Reaction mixtures containing 500 μL of extracts at different concentrations and 3 mL of DPPH solution (24 mg/L in ethanol) prepared fresh were incubated for 30 min at room temperature in the dark. After the incubation period, the absorbance was measured at 517 nm (SPEKOL 1500, Analytik Jena, Jena, Germany). A negative control was prepared using the assay solvent in place of the sample and for the positive control, the sample was replaced with the ascorbic acid standard. The percentage of inhibition of the DPPH radical was calculated according to the following formula and the results are presented as inhibitory concentration (IC_50_), the concentration of the sample required to scavenge 50% of the DPPH radicals:

Inhibition (%)=[(NC Abs−S Abs)⁄NC Abs]×100

where NC Abs is the absorbance of the negative control and S Abs is the absorbance of the sample.

#### 4.7.4. Superoxide Anion Radical Scavenging Assay

The ability of the extracts to scavenge superoxide anion radicals was evaluated according to the method described by Maurício et al., 2024 [86], with some modifications. A reaction mixture containing 200 µL of extract dilutions, 300 μL of β-nicotinamide adenine dinucleotide (NADH) (1.66 mM in phosphate buffer (19 mM, pH 7.4)), 300 μL of nitro blue tetrazolium (NBT) (430 μM in phosphate buffer (19 mM, pH 7.4)), and phosphate buffer (19 mM, pH 7.4) up to 2950 μL was prepared. Then, to initiate the reaction, 50 μL of phenazine methosulphate (PMS) (162 μM) in phosphate buffer (19 mM, pH 7.4) was added and the absorbance at 560 nm (SPEKOL 1500, Analytik Jena, Jena, Germany) was monitored for 2 min at room temperature. A negative control was prepared using the assay buffer in place of the extract sample and for the positive control, the extract sample was replaced by gallic acid. Each extract or gallic acid concentration generated a time-dependent curve, and the slope (ΔAbs/sec) of the linear domain of these curves was used to determine the superoxide anion radical scavenging capacity, as a percentage, according to the following formula:

Inhibition (%)=[(Slope control−Slope sample)⁄Slope control]×100

The percentage of inhibition of the superoxide anion radical was calculated according to the following formula and the results are presented as inhibitory concentration (IC_50_), the concentration of the sample required to scavenge 50% of the superoxide anion radicals generated by the NADH/PMS system:

Inhibition (%)=[(NC Abs−S Abs)⁄NC Abs]×100

where NC Abs is the absorbance of the negative control and S Abs is the absorbance of the sample.

### 4.8. Bovine Serum Albumin Glycation Inhibitory Assay

Bovine serum albumin (BSA) glycation mediated by fructose (FRU) and glucose (GLU) was evaluated according to the methods proposed by Ferron et al., 2020 [87], with slight modifications. Stock solutions of BSA (35 mg/mL), GLU (175 mg/mL), and FRU (175 mg/mL) and freeze-dried samples of the extracts (at different concentrations) were prepared in phosphate buffer (0.1 M, pH 7.4) containing 0.02% (*w*/*v*) sodium azide (to ensure aseptic conditions). Aminoguanidine was used as positive control in both systems and was dissolved in phosphate buffer to obtain 25–400 µg/mL final concentrations in the reaction mixture. Briefly, reaction mixtures containing 100 µL of the BSA solution, 200 µL of the sugar solution (FRU or GLU), and 37.5 µL of sample solutions, aminoguanidine (positive control), or phosphate buffer (negative control) were incubated at 37 °C in an incubator (Mermmet B10, Schwabach, Germany) for 2 days (BSA-FRU system) or 7 days (BSA-GLU). Sample blanks consisting of the samples in phosphate buffer solutions were also incubated at the same time with each BSA-sugar system to evaluate their intrinsic fluorescence. The fluorescence emission of each mixture was measured in a microplate reader (FLUOstar^®^ Omega Plate Reader, Omega V5.70, BMG 264 Labtech, Ortenberg, Germany) with excitation and emission wavelengths of 355 nm and 440 nm, respectively. The percentage inhibition of fluorescent AGE formation (I%) was calculated using the following equation and the results are presented as the inhibitory concentration (IC_50_), the concentration of the sample required to scavenge 50% of fluorescent AGEs:

Inhibition (%)=[1−((S FLU−SB FLU)⁄(NC FLU))]×100

where S FLU is the fluorescence of the sample, SB FLU is the fluorescence of the sample blank, and NC FLU is the fluorescence of the negative control.

### 4.9. Data Analysis

Student’s *t*-test was used for statistical analyses with STATISTICA version 7.0 (StatSoft Inc., Tulsa, OK, USA), and regression analyses were performed with EXCEL version 16.49 (Microsoft corporation, Redmond, WA, USA). The results of antihyperglycemic in vivo assay were presented as the means ± S.E.Ms of *n* observations, where *n* is the number of animals studied. The results were compared using a two-factor ANOVA, followed by Bonferroni’s post hoc test using GraphPad Prism 5.0 software (GraphPad Software Inc., San Diego, CA, USA). *p* < 0.05 was statistically significant.

## 5. Conclusions

*P. chevalieri* aerial part extracts exhibited significant potential to modulate postprandial glycemia in vivo and in vitro. The hydroethanolic extract (170 mg/kg bw) effectively reduced postprandial hyperglycemia in healthy CD1 mice submitted to a sucrose overload. Both aqueous and the hydroethanolic extracts exhibited the potential to delay carbohydrate digestion (inhibition of the α-amylase and α-glucosidase enzymes) in vitro. The results obtained through OSTT and in vitro enzyme inhibition assays (α-glucosidase, α-amylase, and DPP4) provided valuable initial insights into the pharmacological potential of *P. chevalieri*; however, these approaches have inherent limitations concerning translation to animal models or humans. Therefore, future research will aim at performing further in vivo (diabetic animal models) and mechanistic studies (cell systems) necessary to fully elucidate the therapeutic potential of *P. chevalieri,* as well as pharmacokinetic studies and long-term safety assessments.

## Figures and Tables

**Table 1:** HPLC/UV/ESI/MS/MS identification of *P. chevalieri* aerial parts’ main marker secondary metabolites.

Peak	*t*_R_ (min)	UV *λ*_max_ (nm)	[M+H]^+^	[M−H]^−^	MS/MS Fragment Ions *m*/*z* (Relative Abundance)	Proposed Identity	Class
1	6.2	273.2	193	191	191 (31); 127 (15); 93 (27); 87 (26); 85 (100) (25); 59 (22); 45 (24)	Quinic acid	Phenolic acid
2	8.5	279.1	307	305	222 (18), 184 (23), 183 (20), 167 (33), 139 (31), 137(22), 125 (100)	(+)-Gallocatechin	Flavan-3-ol
3	9.6	276.8	595	593	593 (28); 425 (40); 407 (95); 360 (79); 289 (91); 177 (90); 125 (100)	(Epi)gallocatechin (epi)catechin	Proanthocyanidin
4	12.2	276.8	595	593	595 (50); 451(47); 425 (91); 407 (87); 289 (85); 177 (91); 125 (100)	(Epi)gallocatechin (epi)catechin	Proanthocyanidin
5	13.2	278	899	897	897 (11); 611(9); 593 (25);305 (100); 289 (3); 125 (5)	(Epi)gallocatechin (epi)gallocatechin (epi)catechin	Proanthocyanidin
6	18.3	325.3	355	353	191 (100); 179 (75), 161 (5); 155 (4); 135 (41)	3-*O*-caffeoyilquinic acid ^1^ (neochlorogenic acid)	Phenolic acid
7	19.2	280.3; 314.7	579	577	425 (32); 407 (68); 289 (89); 206 (27); 125 (100); 123 (25)	(Epi)catechin (epi)catechin	Proanthocyanidin
8	20.8	279.1; 317.0	579	577	407 (51);385 (100); 289 (45); 223 (40); 125 (52)	(Epi)catechin (epi)catechin	Proanthocyanidin
9	21.6	282.7; 313:5	883	881	881 (18); 611 (15); 593 (22): 577 (11); 515 (5); 305 (100); 289 (3); 125 (4)	(Epi)gallocatechin (epi)catechin (epi)catechin	Proanthocyanidin
10	22.1	280.3	307	305	219 (11), 177 (11), 167 (16), 165 (10), 139 (21), 137(22), 125 (100)	(−)Epigallocatechin	Flavan-3-ol
11	24.8	279.1	291	289	298 (41); 289 (11); 203 (50); 125 (87); 123 (55); 109(100)	(+)-Catechin ^1^	Flavan-3-ol
12	28.9	241; 326.5	355	353	191 (72); 179 (72); 173 (100); 135 (45)	4-*O*-caffeoyilquinic acid (cryptochlorogenic acid)	Phenolic acid
13	30.7	326.5	355	353	191 (100); 179 (2); 173 (2); 161 (1)	5-*O*-caffeoyilquinic acid ^1^ (chlorogenic acid)	Phenolic acid
14	35.8	279.1	291	289	289 (15); 151 (48); 149 (33); 125 (100); 124 (85); 123 (62);109 (74)	(−)-Epicatechin ^1^	Flavan-3-ol
15	47.4	255.6; 355.1	757	755	757 (38); 611 (75); 465, (21); 303 (100)	Quercetin 3-*O*-(2″,6″-di-*O*-rhamnosyl) galactoside	Flavonol
16	47.6	256.7; 355.1	757	755	757 (38); 611 (66); 465, (21); 303 (100)	Quercetin 3-*O*-(2″,6″-di-*O*-rhamnosyl) glucoside	Flavonol
17	50.3	256.7; 356.3	611	609	610 (5) 609(100); 301(11); 300 (12)	Quercetin-3-*O*-rutinoside ^1^ (rutin)	Flavonol
18	50.3	256.7; 356.3	479	477	302(3); 301 (100); 300(2); 179 (3); 151 (5); 113 (4)	Quercetin-3-*O*-glucuronide	Flavonol
19	50.3	256.7; 356.3	465	463	463 (22); 301(72); 300(100);271;227; 151;146	Quercetin-3-*O*-galactoside ^1^ (hyperoside)	Flavonol
20	50.8	328	517	515	353 (100); 179 (58); 173 (82); 191 (17)	1,4-dicaffeoylquinic acid	Phenolic acid

^1^ Compounds identified with commercial reference standard by co-chromatography; MS-MS—mass spectrometry; *m*/*z*—mass-to-charge ratio; *t*_R_—retention time; UV *λ*max·—wavelength of maximum absorbance.

**Table 2:** DER and quantification of the main secondary metabolites in *P. chevalieri* extracts.

Plant Extracts	DER	Phenolic Content (mg GAEs/g)	Flavonoid Content (mg CEs/g)	Condensed Tannin Content (mg CCEs/g)
PcAE	15:1	212.1 ^a^ ± 3.7	122.6 ^a^ ± 3.2	17.43 ^a^ ± 0.09 ^a^
PcEE	2.9:1	415.2 ^b^ ± 1.1	229.1 ^b^ ± 3.1	35.74 ^b^ ± 0.03 ^b^

Abbreviations: PcAE—*Periploca chevalieri* aqueous extract; PcEE—*Periploca chevalieri* hydroethanolic extract; DER—drug–extract ratio; GAEs—gallic acid equivalents; CE—catechin equivalents; CCEs—cyanidin chloride equivalents. In each column, different letters denote significant differences (*p* < 0.05).

**Table 3:** Effects of daily oral administration of the *P. chevalieri* hydroethanolic extract on the body weight and food intake of mice over 13 days.

Parameters	Groups				
	Control (vehicle)	Dose 1 (40 mg/kg)	Dose 2 (170 mg/kg)	Dose 3 (300 mg/kg)	Acarbose (50 mg/kg)
Initial weight (g)	34.2 ± 3.6	34.2 ± 3.5	33.9 ± 5.0	33.6 ± 3.5	34.6 ± 4.2
Final weight (g)	34.9 ± 3.0	35.4 ± 3.4	31.5 ± 3.6	31.9 ± 4.5	34.8 ± 4.2
Body weight variation (%)	1.0 ± 2.7	3.0 ± 1.4 ^#¥^	−3.4 ± 5.8 *	−2.1 ± 1.8	0.7 ± 2.3
Mean food intake per animal (g/day)	5.44 ± 1.96	5.13 ± 1.63	4.4 ± 1.82	4.35 ± 1.66	5.08 ± 1.78

Values are presented as means ± SDs; *n* = 6 to 8; significance level among different groups at *p* ≤ 0.05 (* *p* < 0.05 vs. control; ^#^ *p* < 0.01 vs. PcEE-D2; and ^¥^ *p* < 0.05 vs. PcEE-D3).

**Table 4:** *P. chevalieri* aerial part extracts’ inhibitory activities against the α-amylase, α-glucosidase, and DPP4 enzymes.

Samples	*α*-Amylase IC_50_ (µg/mL)	*α*-Glucosidase IC_50_ (µg/mL)	DPP4 IC_50_ (µg/mL)
PcAE	97.7 ^c^ ± 1.5	17.7 ^b^ ± 0.3	1828.2 ^b^ ± 3.6
PcEE	19.1 ^b^ ± 0.5	7.8 ^a^ ± 0.2	1611.9 ^a^ ± 9.3
Acarbose	11.4 ^a^ ± 0.1	350.3 ^c^ ± 15.3	-
Sitagliptin	-	-	0.0154 ± 0.0003

Abbreviations: DPP4—dipeptidyl peptidase 4; PcAE—*Periploca chevalieri* aqueous extract; PcEE—*Periploca chevalieri* hydroethanolic extract; IC_50_—the half maximal inhibitory concentration. In each column, different letters denote significant differences (*p* < 0.05).

**Table 5:** CUPRAC, FRAP, DPPH^•^ and superoxide anion (O_2_^•−^) radical scavenging capacities of the *P. chevalieri* aerial part extracts.

Samples	CUPRAC (mg AA/g)	FRAP (mg AA/g)	DPPH^•^ IC_50_ (µg/mL)	O_2_^•−^ IC_50_ (µg/mL)
PcAE	438.9 ^a^ ± 3.1	238.1 ^a^ ± 5.1	49.0 ^c^ ± 0.7	15.7 ^b^ ± 1.4
PcEE	941.9 ^b^ ± 38.0	498.1 ^b^ ± 1.5	23.9 ^b^ ± 0.5	7.0 ^a^ ± 1.0
Ascorbic acid	-	-	17.3 ^a^ ± 0.3	-
Gallic acid	-	-	-	16.0 ^c^ ± 3.1

Abbreviations: PcAE—*Periploca chevalieri* aqueous extract; PcEE—*Periploca chevalieri* hydroethanolic extract; CUPRAC—cupric reducing antioxidant capacity, FRAP—ferric reducing antioxidant power, DPPH^•^—2,2-diphenyl-1-picrylhydrazyl radical; O_2_^•−^—superoxide anion; AA—ascorbic acid; IC_50_—the half maximal inhibitory concentration. In each column, different letters denote significant differences (*p* < 0.05).

**Table 6:** *P. chevalieri* aerial part extracts’ antiglycation activities.

Samples	BSA-FRU IC_50_ (µg/mL)	BSA-GLU IC_50_ (µg/mL)
PcAE	23.0 ± 0.5 ^b^	24.3 ± 0.1 ^b^
PcEE	11.0 ± 0.2 ^a^	19.7 ± 0.1 ^a^
Aminoguanidine	136.6 ± 2.1 ^c^	76.2 ± 0.6 ^c^

Abbreviations: BSA—bovine serum albumin; FRU—fructose; GLU—glucose; PcAE—Periploca cheva-lieri aqueous extract; PcEE—*Periploca chevalieri* hydroethanolic extract; IC_50_—the half maximal inhibitory concentration. In each column, different letters denote significant differences (*p* < 0.05).

## Data Availability

The original contributions presented in this study are included in the article. Further inquiries can be directed to the corresponding author.

## Abbreviations

AA, ascorbic acid; AGEs, advanced glycation end products; BSA, bovine serum albumin; CCEs, cyanidin chloride equivalents; CEs, catechin equivalents; CUPRAC, cupric reducing antioxidant capacity; DE, dry extract; DER, drug–extract ratio; DPP4, dipeptidyl peptidase 4; DPPH: 2,2-diphenyl-1-picrylhydrazyl; FRAP, ferric reducing antioxidant power; FRU, fructose; GA, gallic acid; GAEs, gallic acid equivalents; GIP, gastric inhibitory polypeptide/glucose-dependent insulinotropic polypeptide; GLP, glucagon-like peptide-1; GLU, glucose; HDL, high-density lipoprotein; HPLC, high-performance liquid chromatography; HRF, heterocyclic ring fission; LDL, low-density lipoprotein; *m*/*z*, mass-to-charge ratio; MS, mass spectrometry; O_2_^•−^, superoxide anion; PcAE, *Periploca chevalieri* aqueous extract; PcEE, *Periploca chevalieri* hydroethanolic extract; QM, quinone methide; RDA, retro-Diels–Alder; ROS, reactive oxygen species; SGLT2, sodium–glucose co-transporter-2; T2DM, type 2 diabetes mellitus; TCT, total condensed tannin content; TFC, total flavonoid content; TPC, total phenolic content; *t*_R_, retention time; UV, ultraviolet; VLDL, very-low-density lipoprotein; *λ*max, wavelength of maximum absorbance.
